# The environmental impact of community caries prevention - part 2: toothbrushing programmes

**DOI:** 10.1038/s41415-022-4905-3

**Published:** 2022-08-26

**Authors:** Paul Ashley, Brett Duane, Mark Johnstone, Alexandra Lyne

**Affiliations:** 4141510385001 Paediatric Dentistry, Eastman Dental Institute, Rockefeller, 21 University Street, London, WC1E 6DE, UK; 4141510385002 Dental Public Health, Dublin Dental Hospital, Trinity College Dublin, D02 F859, Ireland; 4141510385003 Kent Community Health NHS Foundation Trust, Capital House, Jubilee Way, Faversham, Kent, ME13 8GD, UK

## Abstract

**Introduction** Community-level caries prevention programmes includes supervised toothbrushing in schools and the provision of toothbrushes and toothpaste. The environmental impact of these interventions is an important factor to consider when commissioning these services.

**Materials and methods** A comparative life cycle assessment (LCA) was conducted to quantify the environmental impact of a five-year-old child receiving one of two toothbrushing programmes over a one-year period; supervised toothbrushing in school, or the provision of toothbrushes and toothpaste.

**Results** Supervised toothbrushing had a lower environmental impact than provision of toothbrushes and toothpaste in all 16 impact categories measured. The water use needed for children to brush their teeth was the greatest contributing factor to the provision of toothbrushes and toothpaste, accounting for an average of 48.65% of the impact results.

**Discussion **All community-level caries prevention programmes have an associated environmental cost. LCA is one way to quantify the environmental impact of healthcare services and can be used along with cost and clinical effectives data to inform public healthcare policy. Organisations responsible for these programmes could use the results of this study to consider ways to reduce the environmental impact of their services.

**Supplementary Information:**

Zusatzmaterial online: Zu diesem Beitrag sind unter 10.1038/s41415-022-4905-3 für autorisierte Leser zusätzliche Dateien abrufbar.

## Key points

- This paper is the second in a three-part series looking at the environmental impact of different community-level caries prevention programmes.
- This paper quantifies the environmental impact of two toothbrush-based programmes: supervised toothbrushing in schools and the provision of toothbrushes and toothpaste for use at home.
- This paper analyses what part of these toothbrushing programmes contribute to the environment impact the most.

**Supplementary Information:**

Zusatzmaterial online: Zu diesem Beitrag sind unter 10.1038/s41415-022-4905-3 für autorisierte Leser zusätzliche Dateien abrufbar.

## Introduction

The first paper of this series outlined the urgency of the climate emergency and how health services (including dentistry) need to change in order to become more sustainable. Prevention is the key to any sustainable health delivery. In dentistry, this primarily means caries prevention as dental caries is one of the most common human diseases. This series of papers aims to examine the environmental impact of different types of community-level caries prevention programmes. The first paper focused on fluoride varnish application. This paper considers the sustainability of toothbrushing programmes; supervised toothbrushing in schools and the targeted provision of toothbrushes and toothpaste.

Supervised toothbrushing programmes aim to ensure that children brush their teeth at least once daily during the school day and the targeted provision of toothbrushes and toothpaste programmes aim to ensure that children have access to the resources needed to brush twice daily at home (particularly targeted for children in areas of deprivation or high caries risk). The caries-preventative effect from both of these programmes has been reported^1^^,^^2^ and stems from evidence of brushing with fluoride toothpaste.^3^ Both programmes have been evaluated and are recommended prevention programmes by Public Health England (PHE) and Childsmile in Scotland.^4^^,^^5^

PHE further evaluated the cost effectiveness via a 'return-on-investment' tool; they found that a supervised toothbrushing programmes and targeted provision of toothbrushes and toothpaste are both cost effective, with a return over ten years of £3.66 and £7.34, respectively for every pound spent.^6^

To fully understand any healthcare intervention, the 'triple bottom line' should be evaluated; clinical effectiveness, cost effectiveness and environmental sustainability. While the cost and effectiveness of these programmes have been quantified, their environmental impact has not been researched. The aim of this paper was to quantify the environmental impact of supervised toothbrushing programme in schools and the provision of toothbrushes and toothpaste.

## Materials and methods

The aim of this study was to quantify the environmental impact of toothbrushing schemes using life cycle assessment (LCA) methodology. The functional unit was defined as a single five-year-old child receiving the prevention for one year. The two programmes were:

- Supervised toothbrushing in schools. The dental service provides the materials and training needed to the child's school. School staff supervise the child brushing their teeth for two minutes every school day
- Targeted provision of toothbrushes and toothpaste. The dental service provides toothbrushes and toothpaste, which are delivered to the child via their school. Although the PHE document looks at provision by post or by health visitor, in reality, most community dental programmes deliver the products to children at school, which formed the basis of the model. The assumption is that the child uses these materials to brush their teeth twice daily at home.

The primary outcome was the life cycle impact assessment (LCIA) and secondary outcomes included normalised results, contribution analysis and disability-adjusted life years (DALYs). The secondary aim was to perform a sensitivity analysis to see whether any adjustments to current practice would improve the environmental impact.

The LCA was undertaken at Dublin Dental University Hospital (Trinity College Dublin) in partnership with the University College London Hospitals Eastman Dental Institute, London. An existing community dental service in the UK, along with the Childsmile manual, where used to model the resources needed for each programme.^5^

LCA methodology was applied in line with International Organisation for Standardisation and European Union Product Environmental Footprint (PEF) standards.^7^^,^^8^ The 16 different impact categories used as outcomes and the LCIA methods were based on the PEF guidance and are described in Table 1. The software OpenLCA v1.11 was used alongside the reference database Ecoinvent v3.7.1 for the LCIA and ReCiPe (2016) Endpoint (H) was used to calculate DALYs. The LCIA results were normalised against per capita reference values.

**Table 1:** Impact categories and LCIA methods

Impact category (abbreviation)	LCIA method (units)	Description
Climate change (CC)	IPCC 2013 GWP 100a (kg CO2 eq)	Potential for global warming from greenhouse gas emissions
Ecosystem quality: freshwater and terrestrial acidification (EAC)	ILCD 2011 Midpoint+ (Mol H+ eq)	Acidification of soils and freshwater due to gas release
Ecosystem quality: ecotoxicity freshwater (ECF)	ILCD 2011 Midpoint+ (CTUe)	Harmful effects of toxic substances on freshwater organisms
Ecosystem quality: eutrophication freshwater (EUF)	ILCD 2011 Midpoint+ (kg P eq)	Changes in freshwater organisms and ecosystems caused by excess nutrients
Ecosystem quality: eutrophication marine (EUM)	ILCD 2011 Midpoint + (kg N eq)	Changes in marine organisms and ecosystems caused by excess nutrients
Ecosystem quality: eutrophication terrestrial (EUT)	ILCD 2011 Midpoint + (Molc N eq)	Changes in land organisms from excess nutrients in soil and air
Human health: cancer effects (HCE)	ILCD 2011 Midpoint+ (CTUh)	Harm to human health that causes or increases cancer risk
Human health: ionising radiation (HIR)	ILCD 2011 Midpoint + (kBq U-235 eq)	Potential damage to human DNA from ionising radiation.
Human Health: non-cancer effects (HNC)	ILCD 2011 Midpoint+ (CTUh)	Harm to human health that is not related to cancer or ionising radiation
Human health: respiratory inorganics (HRI)	PM method (Disease inc.)	Harm to human health caused by particulate matter emissions (respiratory disease)
Human health: photochemical ozone formation (HOF)	ILCD 2011 Midpoint + (kg NMVOC eq)	Harm to human health from gas emissions that contribute to smog in the lower atmosphere
Resource use: land use (RLU)	Soil quality index based on LANCA (Pt)	Depletion of natural resources, change in soil quality and reduction in biodiversity
Human health: ozone depletion (HOD)	ILCD 2011 Midpoint+ (kg CFC11 eq)	Air emissions causing stratospheric ozone layer destruction
Resource use: fossils (RFF)	CML-IA baseline (MJ)	Depletion of natural fossil fuels
Resource use: minerals and metals (RMM)	CML-IA baseline (kg Sb eq)	Depletion of natural non-fossil fuel resources
Resource use: dissipated water (RDW)	AWARE (m3 depriv)	Potential for water deprivation to humans and ecosystems globally

**Fig. 1:**
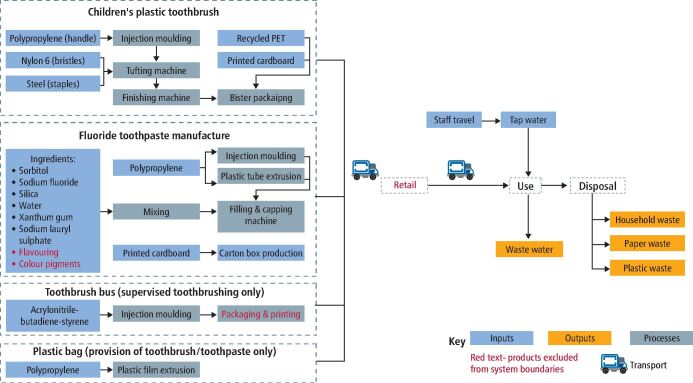
System boundaries for toothbrushing programmes

The system boundaries (resources needed for toothbrushing programmes) are shown in Figure 1. A life cycle inventory was created for each service and is available in the online Supplementary Information. Assumptions and exclusions for each aspect of the life cycle are described below.

### Assumptions for the toothbrushes

Both programmes involved toothbrushes. The supervised toothbrushing in schools programme allocated five toothbrushes per child per school year, to account for lost or dropped toothbrushes. The targeted provision of toothbrushes and toothpaste allocated four toothbrushes per child per year (one pack containing one toothbrush given every three months).

A life cycle inventory for a manual, plastic, children's size toothbrush was created from a teardown of a sample product. Detailed assumptions about the manufacture, packing and transport are described in a previous LCA study of toothbrushes.^9^ In brief, a polypropylene handle was made via injection moulding and nylon bristles were stapled into the brush head and polished using a tufting and finishing machine. Using a blister packaging machine, the toothbrush was then packaged in polyethylene terephthalate and printed cardboard. The location of manufacture and packaging was assumed to be in Europe, with toothbrushes then transported via lorry and ferry to the population centre of the UK.^10^ For disposal of the product, it was assumed the packaging was recycled and the toothbrush itself thrown in the bin.

For the purpose of sensitivity analysis, the life cycle inventory for a children's manual bamboo toothbrush was created from a teardown of a sample children's toothbrush product and based on assumption from the same previous toothbrush LCA.^9^ In brief, the raw bamboo was assumed to be grown in China, with bamboo transported to a nearby factory for manufacture via wood shaping machinery and infrared heat treatment. Nylon bristles and brass staples were inserted into the head using tufting and finishing machinery and then packaged in a single printed cardboard box. This was then transported to the UK population centre via sea and land transport. The toothbrush was assumed to be thrown in in the bin and the cardboard packaging recycled.

### Assumptions for the toothpaste

Both programmes used fluoride toothpaste. Based on the Childsmile manual, for supervised toothbrushing in schools, it was assumed that one child would use 0.65 tubes of toothpaste per year, based on a 'pea-sized' amount of toothpaste.^11^ However, there is evidence that adults will dispense more than a 'pea-sized' amount of toothpaste. Creeth *et al.* found that UK adults actually dispensed twice the amount of the reference 'pea-sized' amount.^12^ Therefore, a sensitivity analysis was performed in this LCA using this higher estimate for toothpaste (equating 1.3 tubes of toothpaste per child per year, instead of 0.65). The targeted provision of toothbrushes and toothpaste allocated four toothpaste tubes per child per year (one pack containing one tube of 100 ml toothpaste given every three months).

A life cycle inventory was created for a single tube of fluoride toothpaste. The toothpaste was assumed to come in 100 ml tubes and contain 1,450 ppm sodium fluoride. The following toothpaste ingredients and proportions were based on a generic 'recipe' for toothpaste, as described by Vranic *et al.* (2004):^13^

- Sodium fluoride (active ingredient) - 0.315%
- Silicia (abrasive) - 25%
- Xanthan gum (binder) - 1.5%
- Sorbitol (humectant) - 53.66%
- Water (solvent) - 17.5%
- Sodium benzoate (preservative) - 0.275%
- Sodium lauryl sulphate (foaming agent) - 1.75%.

Flavourings and colourants were excluded as they vary between manufacturers and are present in small amounts. Following interview with a toothpaste manufacturer, it was assumed a 68 kW, 500 L barrel toothpaste mixing machine was used to mixed toothpaste ingredients over 2.4 hours. The kilowatt hours (kWh) needed to produce 100 ml of toothpaste gel was included; however, cleaning and maintenance of this machinery was excluded from the system boundaries.

Packaging of the toothpaste was based on a teardown of a sample product. The mixed toothpaste was placed in high-density polyethylene tube, which was then sealed and capped. This tube was placed in a printed cardboard box. The kWh of the filling, sealing and capping machinery was used to model the manufacturing processes, along with the raw materials. It was assumed the toothpaste was manufactured in Europe and shipped to the UK population centre by road and sea transport. It was assumed the cardboard packaging was disposed of in cardboard recycling and the empty tube of toothpaste in the bin. The excess toothpaste at the end of brushing was assumed to be washed down the drain along with the tap water from the toothbrushing.

### Assumptions of tap water

It was assumed that each child would use 2 L of tap water each time they brush their teeth. This is based on the average UK tap using 6 L per minute^14^ and assuming the supervising adult has the tap running for a total of 20 seconds (time to wash the toothbrush before and after brushing but turning the tap off during the two minutes of brushing).

For the supervised toothbrushing programme, it was assumed the child would attend school for all 195 days in the calendar year doing supervised toothbrushing every day; therefore, using 390 L of tap water per year.^15^ Children in the supervised toothbrushing programmes may have also been practising toothbrushing at home but the resources needed for this were excluded as it was not part of the supervised toothbrushing programme itself.

For the provision of toothbrushes and toothpaste, the expectation of providing these packs is that a child who was previously unable to brush their teeth at home would then be able to brush twice daily every day for 365 days, which would equate to 1,460 L of tap water per year. However, there is no evidence on how many 'extra' episodes of toothbrushing a child does after receiving these packs. Therefore, to understand the environmental impact of this prevention programme more robustly, the full amount tap water was included in the original model and then excluded in a sensitivity analysis.

### Other assumptions

The supervised toothbrushing programme used a toothbrush holder (often shaped as a bus or a train, hence the term 'toothbrush bus') to store the children's toothbrushes between uses. This toothbrush bus was replaced each term, That is, three are used per year. Following an interview of a UK-based manufacturer of a toothbrush bus, it was confirmed the ten-toothbrush bus holder was made from 177 g of acrylonitrile-butadiene-styrene, which was formed via injection moulding. Printing, packaging and colourings were excluded for the toothbrush bus as this varies between manufacturers. The bus was assumed to be shipped, unpackaged, from the manufacturing location to the UK population centre via road and sea transport.^10^

The provision of toothbrushes and toothpaste did not require any toothbrush holder; however, it did require a plastic zip lock bag to hold the toothbrush and toothpaste. The plastic bag was assumed to be made from extruded polypropylene plastic film, weighing 1.5 g and was disposed of in plastic recycling.

For both programmes, it was assumed that the resources needed (toothbrushes, toothpaste, toothbrush buses or plastic bags) were delivered to a central dental centre (assumed to be located in the UK population centre)^10^ and subsequently delivered to the child's school (along with other support and training for school staff) every three months. As in the first paper of this series, it was assumed a single member of dental staff would travel from the dental centre, driving a small van (EUR05 engine) and cover two schools in a day, equating to 180 children. The round-trip journey to two schools was estimated as 13 km, based on the distances travelled by one existing programme in England, assuming two geographically close schools are selected for each trip.

## Results

The results of the LCIA are shown in Tables 2 and 3, along with the results of the sensitivity analyses. Supervised toothbrushing in schools produced 1.95 kg of carbon, compared to 2.89 kg for the provision of toothbrushes and toothpaste.

**Table 2:** LCIA results for supervised toothbrushing and sensitivity analysis

Impact category (units)	Supervised toothbrushing	Sensitivity analysis - using bamboo instead of plastic toothbrushes	Sensitivity analysis - using 0.5 g of toothpaste to brush teeth
Climate change (kg CO2 eq)	1.95E+00	1.66E+00	2.08E+00
Acidification (mol H+ eq)	8.11E-03	7.18E-03	8.75E-03
Freshwater ecotoxicity (CTU)	3.22E+00	2.86E+00	3.25E+00
Freshwater eutrophication (kg P eq)	5.30E-04	3.80E-04	5.80E-04
Marine eutrophication (kg N eq)	1.93E-03	1.88E-03	2.17E-03
Terrestrial eutrophication (mol N eq)	1.75E-02	1.64E-02	1.85E-02
Carcinogenic effects (ctuh)	1.04E-07	9.53E-08	1.04E-07
Ionising radiation (kg U235 eq)	1.41E-01	1.09E-01	1.40E-01
Non-carcinogenic effects (ctuh)	1.96E-07	1.89E-07	2.06E-07
Ozone layer depletion (kg CFC-11 eq)	3.92E-07	1.27E-07	3.82E-07
Photochemical ozone creation (kg NMVOC eq)	5.97E-03	5.37E-03	6.32E-03
Respiratory inorganics effects (disease inc)	9.02E-08	8.97E-08	1.08E-07
Dissipated water (m3 water eq)	2.43E+00	2.32E+00	2.66E+00
Fossil use (MJ)	3.19E+01	2.44E+01	3.50E+01
Land use (pts)	1.17E+01	1.96E+01	1.25E+01
Mineral/metal use (kg Sb eq)	1.79E-05	1.38E-05	1.70E-05

**Table 3:** LCIA results for provision of toothbrushes and toothpaste and sensitivity analysis

Impact category (units)	Targeted provision of toothbrushes and toothpaste	Sensitivity analysis - using bamboo instead of plastic toothbrush	Sensitivity analysis - using paper instead of plastic bag	Sensitivity analysis - using bamboo toothbrush AND paper bag	Sensitivity analysis - excluding water use
Climate change (kg CO2 eq)	2.89E+00	2.72E+00	2.87E+00	2.71E+00	1.85E+00
Acidification (mol H+ eq)	1.36E-02	1.32E-02	1.36E-02	1.31E-02	8.33E-03
Freshwater ecotoxicity (CTU)	5.28E+00	5.21E+00	5.29E+00	5.21E+00	2.28E+00
Freshwater eutrophication (kg P eq)	1.06E-03	9.60E-04	1.07E-03	9.60E-04	5.40E-04
Marine eutrophication (kg N eq)	3.70E-03	3.72E-03	3.71E-03	3.73E-03	2.50E-03
Terrestrial eutrophication (mol N eq)	2.80E-02	2.79E-02	2.81E-02	2.80E-02	1.64E-02
Carcinogenic effects (ctuh)	2.59E-07	2.56E-07	2.59E-07	2.56E-07	4.38E-08
Ionising radiation (kg U235 eq)	2.40E-01	2.19E-01	2.40E-01	2.20E-01	6.19E-02
Non-carcinogenic effects (ctuh)	4.51E-07	4.53E-07	4.52E-07	4.53E-07	1.50E-07
Ozone layer depletion (kg CFC-11 eq)	3.33E-07	1.34E-07	3.33E-07	1.35E-07	2.67E-07
Photochemical ozone creation (kg NMVOC eq)	9.24E-03	9.02E-03	9.24E-03	9.02E-03	5.61E-03
Respiratory inorganics effects (disease inc)	2.28E-07	2.31E-07	2.28E-07	2.31E-07	1.44E-07
Dissipated water (m3 water eq)	8.37E+00	8.29E+00	8.37E+00	8.28E+00	1.58E+00
Fossil use (MJ)	5.19E+01	4.69E+01	5.16E+01	4.65E+01	3.57E+01
Land use (pts)	2.08E+01	2.77E+01	2.42E+01	3.12E+01	1.21E+01
Mineral/metal use (kg Sb eq)	1.87E-05	1.65E-05	1.87E-05	1.64E-05	9.46E-06

The sensitivity analysis for the supervised toothbrushing programme shows that swapping the plastic children's toothbrushes for bamboo children's toothbrushes results in a reduction in the impact in all but one category (land use, which increased by 68% when bamboo toothbrushes were used). The climate change impact is reduced by 15%; freshwater eutrophication by 28%; mineral and metal use by 23%; and water use by 5%. Using a realistically dispensed 'pea-sized' amount of toothpaste (0.5 g per toothbrushing episode rather than 0.25 g) increased the impact result in 12 out of 16 categories, between 1% (freshwater ecotoxicity) and 19% (respiratory inorganic effects).

Sensitivity analysis for the provision of toothbrushes and toothpaste also looked at using bamboo toothbrushes, as well as paper instead of plastic bags. All these sensitivity analyses scenarios reduced the LCIA but only by a small amount. Using climate change as an example, using bamboo toothbrushes reduced the impact by 6%, using paper bags reduced the impact by 0.47% and using both bamboo toothbrushes and paper bags reduced the impact by 6.1%. The final sensitivity analysis for this programme excluded the water used by children to brush their teeth at home; this found a vastly reduced impact result in all categories (for example, climate change reduced by 36%, water use by 81% and freshwater eutrophication by 49%).

**Fig. 2:**
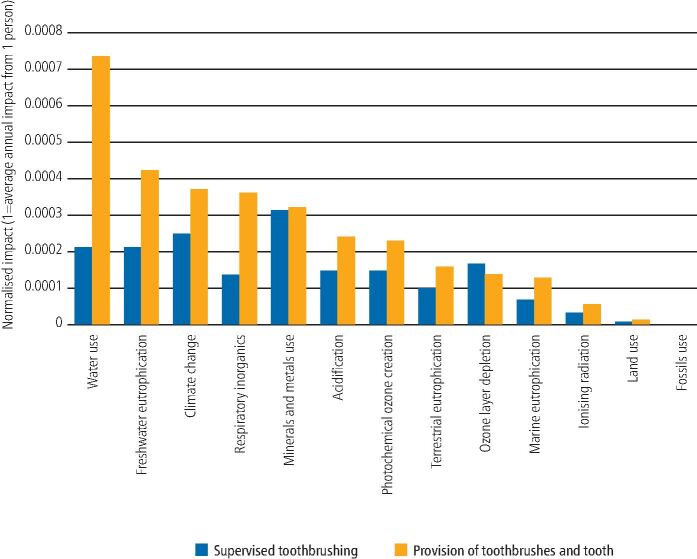
Normalised results for toothbrushing programmes

The LCIA results for the original models were normalised against average global reference values for the annual environmental footprint of the average person, as shown in Figure 2. Following PEF recommendations, the three toxicity-related categories have been excluded while the robustness of the methodology is under review.^8^ Mineral and metal use was the most important impact category for the supervised toothbrushing programme, using the equivalent of 0.03% of the average person's mineral and metal resource use in one year. For the provision of toothbrushes and toothpaste (which included the water used by the child to brush their teeth twice daily at home), the water use was the most important impact category, using the equivalent of 0.07% of the average person's water use in one year.

**Fig. 3:**
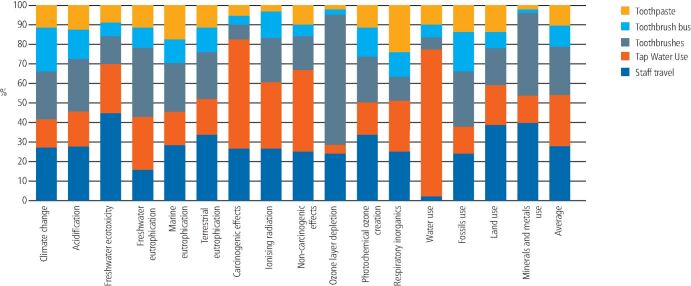
Contribution analysis for supervised toothbrushing

**Fig. 4:**
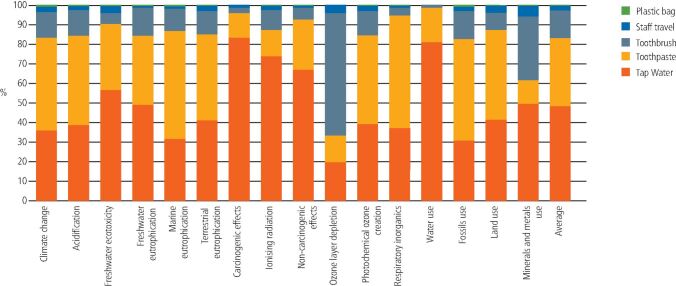
Contribution analysis for provision of toothbrushes and toothpaste

Figures 3 and 4 show the contribution analysis for each programme. For supervised toothbrushing, staff travel (one member of the dental team travelling into their place of work, then driving the materials to the schools) was the greatest overall contributor to the LCIA results (average 28.39%; range 2.85-45.23%), followed closely by the tap water use and waste (average 25.92%; range 4.51-74.57%) and the plastic toothbrushes (average 24.52%; range 6.14-65.98%). The toothbrush bus and the toothpaste itself were the lowest contributors (an average of 10.79% and 10.38%, respectively).

For the provision of toothbrushes and toothpaste, the water use was the greatest contributor, which contributed 48.65% on average (range from 19.98% in ozone layer depletion to 81.18% in water use), followed by the toothpaste, which contributed an average of 34.61% (range from 12.16% in mineral and metal use to 57.24% in respiratory inorganic effects). The plastic toothbrush contributed an average of 13.86% to the overall result, with its highest contribution at 62.19% for ozone depletion.

When looking at a single 100 ml tube of 1,450 ppm sodium fluoride toothpaste, the greatest contributing factor within the toothpaste itself is the sorbitol, which accounted for an average of 48.2% of the toothpaste contribution (range 1.84-85.24%). Other highest contributing elements of the toothpaste included transport, plastic tube and mixing machinery (accounting for an average of 11.41%, 10.38% and 10.21%, respectively).

Table 4 shows the DALY calculations. The DALY impact was 114 seconds for supervised toothbrushing and 279 seconds for provision of toothbrushes and toothpaste. For both programmes, over 99.99% of the DALY impact came from global warming and water consumption, with all other human health impact categories contributing less than 0.01% to the result.

**Table 4:** DALYs for toothbrushing programmes

Human health impact category	Supervised toothbrushing	Provision of toothbrushes and toothpaste
Global warming	1.81359E-06	2.6799E-06
Stratospheric ozone depletion	2.0815E-10	1.7668E-10
Ionising radiation	1.19944E-09	2.0407E-09
Particulate matter formation	5.67928E-11	1.4382E-10
Photochemical ozone formation	5.23136E-09	8.0968E-09
Cancer effects	3.44848E-13	8.5907E-13
Non-cancer effects	1.30085E-15	3.0017E-15
Water consumption	1.78949E-06	6.1534E-06
Total DALYs	3.60978E-06	8.8438E-06
DALY seconds	113.8380508	278.898726

## Discussion

This is the first result paper that quantifies the environmental impact of a preventive programme involving the provision of toothbrushes and toothpaste. This study relied on LCAs to determine the sustainability of the interventions assessed. As with any LCA there are a number of assumptions that need to be made in order to generate the final values.

In this study, the programmes from two existing public health programmes were modelled. Information about the resources needed for each programme were based on the Childsmile manual and interview with our own service in England.^5^^,^^11^ Admittedly, those details, such as the travel distances to and from schools, were specific to the geography of each service and may not apply to other parts of the world. The intention of this paper is that the description of both input and outputs provide a way for service planners to consider the applicability of these results to their unique programmes.

The toothpaste was modelled as a generic toothpaste with minimal ingredients required. This was not based on a specific brand and there is likely to be variation between manufacturers. At present, attempting to determine the detailed life cycle processes of a particular brand is not possible if manufacturers are unwilling to divulge the information. As argued previously, given the importance of sustainability, the responsibility should lie with toothpaste manufacturers to quantify and improve the impact of their products.^9^ This will help drive innovation in to ways to reduce impact and support further assessments of sustainability studies.

As with the first paper in this series, DALYs are reported in the results. Again, these should be interpreted with caution due to the assumptions made. However, these figures are useful to not only consider planetary health impacts (for example, carbon dioxide equivalent emissions), but also to consider the human health effects of clinical interventions.

Based on the models in this paper, supervised toothbrushing performs better from an environmental perspective than provision of toothbrushes and toothpaste. This is not surprising, since a supervised toothbrushing programme includes 195 episodes of toothbrushing (school days in a year), whereas a child brushing twice daily at home would equate to 730 episodes of toothbrushing. However, before drawing firm conclusions from this, it is important to consider the assumptions made. For supervised toothbrushing, the model did not include any of the resources, materials, or water that the child might be already using for toothbrushing at home (it only accounted for the 195 episodes of brushing). For the provision of toothbrushes and toothpaste, the assumption was that the child would go from zero episodes of toothbrushing at home to 730 episodes of brushing, as a result of the programme. However, providing access to toothbrushes and toothpaste does not necessarily mean that the children would brush their teeth twice a day and there is no evidence available for how many additional episodes of toothbrushing happen as a direct result of this programme. Until there is further evidence available on the compliance with twice daily toothbrushing at home, it is difficult to conclude that one is more sustainable than the other, which is why a sensitivity analysis was done excluding any water use at all. Unsurprisingly, this lowered the environmental impact significantly, but if the child is not using the toothbrushes and toothpaste with water at home, then they are not getting the associated caries preventative effect.

There are a number of ways that both of these interventions could be made more environmentally sustainable. There are considerable environmental savings to be made by using less water ie dry brushing, with a small amount of water used to wash the bristles of the brush. However, this may not be acceptable to children. PHE updated guidance for supervised toothbrushes programmes during the COVID-19 pandemic and recommended dry brushing to reduce water droplet transmission of the virus.^16^

Swapping to bamboo toothbrushes improves climate change impact but only by a modest amount - 15% for supervised toothbrushing and 6% for provision of toothbrushes and toothpaste. This swap, however, does increase the land use impact, which will have a knock-on negative effect on biodiversity. Bamboo as an alternative to plastic in toothbrushes and other products is a positive step but is not the ideal answer.

A previous LCA study of hypothetical toothbrush designs found that toothbrush handles made from recycled polypropylene toothbrush handles are the most sustainable toothbrush model; however, at the time of conducting this study, a product that follows this model is not currently available on the UK market and plastic recycling in reality is rarely as efficient as it could be.^17^ However, with demand for 'eco-friendly' products, toothbrush manufacturers are constantly bringing new products to market, including products that use recycled plastic. The assumption in this study was that the toothbrush is thrown in normal waste. Although Childsmile advocates that plastic toothbrushes can be disposed of in plastic recycling, there is no evidence on whether individuals do this and how much plastic UK households place in plastic waste actually gets recycled.^18^ Private recycling companies, such as Terracycle, claim to use recycled toothbrushes to make other plastic products but to our knowledge, the environmental consequences of this process has not been evaluated via a LCA process.^19^ The use of new plastic recycling programmes using pyrolysis may lead to a significant reduction in environmental costs and may assist in better plastic reduction.^20^^,^^21^ Whichever recycling scheme is finally used, the microbiological safety of it will need to be established.

The fluoride toothpaste was a contributor to the environmental footprint of both supervised toothbrushing (10.38%) and provision of toothbrushes and toothpaste (34.61%). Sub-analysis of the toothpaste tube found that this impact was mainly due to the sorbitol ingredient, which forms the majority of the weight of the toothpaste. It may be possible to come up with a more sustainable sweetener than sorbitol, produce products locally, or use more sustainable transport solutions. There are a number of companies who are now offering toothpaste containers where this exists locally.^22^ There may be more sustainable options, for example, providing chewable fluoride toothpaste 'tablets', which have been shown to have comparable fluoride bioavailability to traditional toothpaste;^23^ however, more research is needed to understand both the clinical and environmental effects before recommending toothpaste tablets.

For supervised toothbrushing, staff travel to and from work to deliver materials was a big contributor - this reinforces that it is not just the materials needed for a healthcare programme that matter; there is individual responsibility of staff to consider the environmental impact of their travel to work and responsibility of employers and essentially government to encourage and facilitate this.

## Conclusion

Both toothbrushing programmes were associated with an environmental impact. Before recommending one programme over another, more research is needed about the provision of toothbrushes and toothpaste programmes and how many additional episodes of toothbrushing at home the child actually complies with. Both programmes showed a modest improvement in climate change impact when bamboo toothbrushes were used instead of plastic toothbrushes.

## Supplementary Information

Supplementary Information (PDF 100KB)
