# Supplementary material for: The environmental impact of community caries prevention - part 2: toothbrushing programmes
Source: Br Dent J. 2022 Aug 26;233(4):295–302. doi: 10.1038/s41415-022-4905-3 (PMC9412791; doi:10.1038/s41415-022-4905-3)
Supplement: Supplementary file 1 — Supplementary Information (PDF 100KB) [file 41415_2022_4905_MOESM1_ESM.pdf]

ONLINE SUPPLEMENTARY MATERIAL

Life cycle inventory tables for supervised toothbrushing in schools & provision of toothbrushes and toothpaste

Flow	Amount	Unit	Provider
Supervised toothbrushing in schools<br>**0.65 was changed to 1.3 for sensitivity analysis*<br>***Children's plastic toothbrush was changed to childrens bamboo toothbrush for sensitivity analysis*			
Tubes of toothpaste	0.65*	Item(s)	1450 toothpaste (see below)
Material for toothbrush bus	0.0531	kg	market for acrylonitrile-butadiene-styrene copolymer | acrylonitrile-butadiene-styrene copolymer | Cutoff, U - GLO
Toothbrush	5	Item(s)	Childrens plastic toothbrush (see below)**
Manufacture for toothbrush bus	0.0531	kg	injection moulding | injection moulding | Cutoff, U - RER
Staff travel to work	0.02222222	Item(s)	Staff travel one return journey
tap water	390	kg	market for tap water | tap water | Cutoff, U - Europe without Switzerland
Land transport for toothbrush bus	14.84676	kg*km	transport, freight, lorry, all sizes, EURO6 to generic market for transport, freight, lorry, unspecified | transport, freight, lorry, unspecified | Cutoff, U - RER
Sea transport for toothbrush bus	11.5758	kg*km	transport, freight, sea, ferry | transport, freight, sea, ferry | Cutoff, U - GLO
Staff travel to schools	0.28888889	km	transport, passenger car, EURO 5 | transport, passenger car, EURO 5 | Cutoff, U - RER
Disposal of toothbrush bus	0.0531	kg	market for waste polystyrene | waste polystyrene | Cutoff, U - GB
Waste tap water	390	l	market for wastewater, unpolluted, from residence | wastewater, unpolluted, from residence | Cutoff, U - RoW

Targeted provision of toothbrushes and toothpaste<br>**Children's plastic toothbrush was changed for children's bamboo toothbrush for sensitivity analysis*<br>***Plastic bag flows were changed for a cardboard bag for sensitivity analysis (8.12g allocated, providers were market for paper sack, Cutoff, U – RER and market for waste paperboard, Cutoff, U – GB)*			
1450 toothpaste	4	Item(s)	1450 toothpaste, see below
Childrens toothbrush	4	Item(s)	Children's plastic toothbrush, see below*
Manufacture of plastic bag**	6	g	extrusion, plastic film | extrusion, plastic film | Cutoff, U - RER

Material for plastic bag**	6	g	market for polypropylene, granulate | polypropylene, granulate | Cutoff, U - GLO
tap water	1460	kg	market for tap water | tap water | Cutoff, U - Europe without Switzerland
Delivery of items	0.23423423	km	transport, passenger car, EURO 5 | transport, passenger car, EURO 5 | Cutoff, U - RER
Waste plastic bag**	6	g	market for waste polypropylene | waste polypropylene | Cutoff, U - GB
Waste water	1460	l	market for wastewater, unpolluted, from residence | wastewater, unpolluted, from residence | Cutoff, U - RoW

1 tube of 1450 sodium fluoride toothpaste			
sodium fluoride	0.3142125	g	market for sodium fluoride | sodium fluoride | Cutoff, U - GLO
silica	24.9375	g	market for silica sand | silica sand | Cutoff, U - GLO
sorbitol	53.52585	g	market for polyol | polyol | Cutoff, U - RER
water	17.45625	g	market for water, deionised | water, deionised | Cutoff, U - Europe without Switzerland
xanthum gum	1.49625	g	market for carboxymethyl cellulose, powder | carboxymethyl cellulose, powder | Cutoff, U - GLO
benzoic acid	0.13715625	g	market for benzoic acid | benzoic acid | Cutoff, U - RER
SLS	0.13715625	g	market for neutralising agent, sodium hydroxide-equivalent | neutralising agent, sodium hydroxide-equivalent | Cutoff, U - GLO
alkyl sulphate (C12-14)	1.745625	g	market for alkyl sulphate (C12-14) | alkyl sulphate (C12-14) | Cutoff, U - GLO
mixing machinery	0.03264052	kWh	market for electricity, medium voltage | electricity, medium voltage | Cutoff, U - PL
filling and capping tube	0.00222222	kWh	market for electricity, medium voltage | electricity, medium voltage | Cutoff, U - PL
printed cardboard box	13.1	g	market for carton board box production, with offset printing | carton board box production, with offset printing | Cutoff, U - GLO

plastic for tube lid	4.91	g	market for polypropylene, granulate | polypropylene, granulate | Cutoff, U - GLO
manufacture of tube lid	4.91	g	injection moulding | injection moulding | Cutoff, U - RER
plastic for tube	4.84	g	market for polyethylene, high density, granulate | polyethylene, high density, granulate | Cutoff, U - GLO
manufacture of tube	4.84	g	extrusion, plastic pipes | extrusion, plastic pipes | Cutoff, U - RER
Land transport to UK	173.36866	kg*km	transport, freight, lorry, all sizes, EURO6 to generic market for transport, freight, lorry, unspecified | transport, freight, lorry, unspecified | Cutoff, U - RER
Sea transport to UK	6.13	kg*km	market for transport, freight, sea, ferry | transport, freight, sea, ferry | Cutoff, U - GLO
Disposal of tube/lid	13.1	g	market for municipal solid waste | municipal solid waste | Cutoff, U - GB
Disposal of cardboard packaging	9.75	g	market for waste paperboard | waste paperboard | Cutoff, U - GB
1 children's plastic toothbrush			
plastic handle material	11.16	g	market for polypropylene, granulate | polypropylene, granulate | Cutoff, U - GLO
bristle material	0.85	g	market for nylon 6 | nylon 6 | Cutoff, U - RER
staple material	0.08	g	market for steel, low-alloyed | steel, low-alloyed | Cutoff, U - GLO
manufacture of handle	14.41	g	injection moulding | injection moulding | Cutoff, U - RER
manufacture of bristle head	0.0029525	kWh	market for electricity, medium voltage | electricity, medium voltage | Cutoff, U - PL
manufacture to finish bristle head	0.000476	kWh	electricity voltage transformation from high to medium voltage | electricity, medium voltage | Cutoff, U - PL
cardboard packaging material	2.93	g	market for carton board box production, with offset printing | carton board box production, with offset printing | Cutoff, U - GLO
plastic packaging material	3.25	g	market for polyethylene terephthalate, granulate, bottle grade | polyethylene terephthalate, granulate, bottle grade | Cutoff, U - GLO

manufacture of blister packaging	0.011905	kWh	market for electricity, medium voltage | electricity, medium voltage | Cutoff, U - PL
Sea transport to UK	17.59401	kg*km	transport, freight, sea, ferry | transport, freight, sea, ferry | Cutoff, U - GLO
Land transport to UK	0.787437	kg*km	transport, freight, lorry, all sizes, EURO6 to generic market for transport, freight, lorry, unspecified | transport, freight, lorry, unspecified | Cutoff, U - RER
Disposal of toothbrush	12.09	g	market for municipal solid waste | municipal solid waste | Cutoff, U - GB
Disposal of cardboard packaging	2.93	g	market for waste paperboard | waste paperboard | Cutoff, U - GB
Disposal of plastic packaging	3.25	g	market for waste polyethylene | waste polyethylene | Cutoff, U - GB
1 children's bamboo toothbrush			
Bamboo handle material	7.91	g	Bamboo cultivation - CN
Bristle material	0.55	g	market for nylon 6 | nylon 6 | Cutoff, U - RoW
Staple material	0.08	g	market for brass | brass | Cutoff, U - RoW
Manufacture of bamboo handle (wood shaping machine)	0.01022515	kWh	electricity voltage transformation from high to medium voltage | electricity, medium voltage | Cutoff, U - CN-SGCC
Manufacture of bamboo handle (heat treatment of bamboo)	0.0108335	kWh	electricity voltage transformation from high to medium voltage | electricity, medium voltage | Cutoff, U - CN-SGCC
manufacture of bristle head	0.0029525	kWh	electricity voltage transformation from high to medium voltage | electricity, medium voltage | Cutoff, U - CN-SGCC
manufacture to finish bristle head	0.000476	kWh	electricity voltage transformation from high to medium voltage | electricity, medium voltage | Cutoff, U - CN-SGCC
Cardboard packaging material	8.77	g	market for carton board box production, with offset printing | carton board box production, with offset printing | Cutoff, U - GLO
Land transport to UK	13.98645	kg*km	transport, freight, lorry, all sizes, EURO6 to generic market for transport, freight, lorry, unspecified | transport, freight, lorry, unspecified | Cutoff, U - RoW

Sea transport to UK	372.27471	kg*km	transport, freight, sea, container ship | transport, freight, sea, container ship | Cutoff, U - GLO
Disposal of toothbrush	8.54	g	market for municipal solid waste | municipal solid waste | Cutoff, U - GB
Disposal of cardboard packaging	8.77	g	market for waste paperboard | waste paperboard | Cutoff, U - GB
